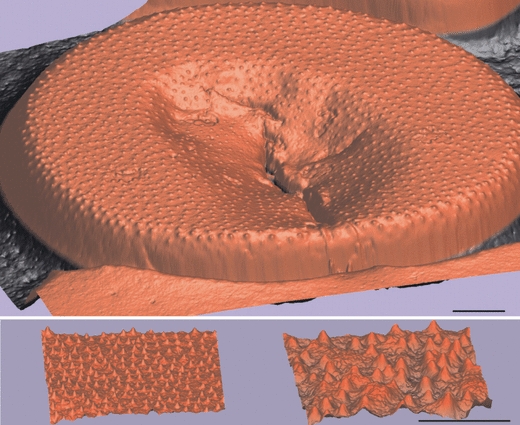# High density of ‘spiky’ excrescences covering the surface of an erythrocyte infected with *Plasmodium malariae*

**DOI:** 10.1111/j.1365-2141.2010.08261.x

**Published:** 2010-10

**Authors:** Ang Li, Bruce Russell, Laurent Renia, Usa Lek-Uthai, Francois Nosten, Chwee T Lim

**Affiliations:** 1 Nano Biomechanics Laboratory, Division of Bioengineering and Department of Mechanical Engineering, National University of SingaporeSingapore; 2 Singapore Immunology Network (SIgN), A*STARBiopolis, Singapore; 3 Department of Parasitology and Entomology, Faculty of Public Health, Mahidol UniversityBangkok, Thailand; 4 Shoklo Malaria Research UnitMae Sod, Thailand; 5 Nuffield Department of Clinical Medicine, University of OxfordCCVTM, Oxford, UK; 6 Faculty of Tropical Medicine, Mahidol UniversityRajvithi Road, Bangkok Thailand

This Atomic Force Microscope image shows the surface of a *Plasmodium malariae* trophozoite-infected red blood cell isolated from a Burmese patient suffering from quartan malaria (top). Of note, the *P. malariae*-infected cell (bottom left) is covered with dense ‘spike-like’ excrescences (mean height: 7·59 nm; mean diameter: 52·95 nm), which are morphologically distinct from the larger, more rounded ‘knob’ structures found on a *Plasmodium falciparum-*infected red cell (mean height: 19·65 nm; mean diameter: 96·64 nm) (bottom right). The ‘knobs’ on red cells containing mature asexual forms of *P. falciparum* assist the infected cells to bind/sequester to the vascular endothelium under shear flow conditions and thus avoid splenic clearance. The function of *P. malariae* spikes (which we have observed on every sexual and asexual stage examined in isolates from Indonesia and Thailand) is not known. The sample from which the *P. malariae* pictured was isolated (Mae Sod, Tak, Thailand) had the diagnosis confirmed by polymerase chain reaction. This picture is artificially coloured. The horizontal black scale bars represent 1 μm. The two lower images are identical in scale and orientation.

**Figure:**